# EPidemiology, clinical characteristics and Outcomes of 4546 adult admissions to high-dependency and intensive care units in Kenya (EPOK): a multicentre registry-based observational study

**DOI:** 10.1097/CCE.0000000000001036

**Published:** 2024-02-01

**Authors:** Carolyne Njoki, Nabukwangwa Simiyu, Ronnie Kaddu, Wambui Mwangi, Demet Sulemanji, Peter Oduor, Dilanthi Gamage Dona, Dorothy Otieno, Teddy Thaddeus Abonyo, Patricia Wangeci, Thomas Kabanya, Selina Mutuku, Annastacia Kioko, Joy Muthoni, Peter Mburu Kamau, Abigail Beane, Rashan Haniffa, Arjen Dondorp, David Misango, Luigi Pisani, Wangari Waweru-Siika

**Affiliations:** 1 Department of Anesthesia, Aga Khan University, Nairobi, Kenya; 2 Department of Anesthesia and Intensive Care, Kisii Hospital, Kisii, Kenya; 3 Intensive Care Unit, Aga Khan Mombasa Hospital (AKM), Mombasa, Kenya; 4 Intensive Care Unit, Nyeri County Hospital, Nyeri, Kenya; 5 Department of Anesthesia and Intensive Care, MP Shah Hospital, Nairobi, Kenya; 6 Department of Anesthesia and Intensive Care, Nakuru referral Hospital, Nakuru, Kenya; 7 Nat Intensive Care Surveillance-MORU, Colombo, Sri Lanka; 8 Critical Care Society of Kenya, Nairobi, Kenya; 9 Mahidol Oxford Tropical Research Unit, Bangkok, Thailand

**Keywords:** Intensive care unit, Kenya, high dependency unit, outcomes, registry

## Abstract

**Objective:**

to describe clinical, management and outcome features of critically ill patients admitted to intensive care units (ICUs) and high dependency units (HDUs) in Kenya.

**Design:**

prospective registry-based observational study.

**Setting:**

three HDUs and eight ICUs in Kenya.

**Patients:**

consecutive adult patients admitted between January 2021 and June 2022.

**Interventions:**

none.

**Measurements and main results:**

data was entered in a cloud based platform using a common data model. Study endpoints included case mix variables, management features and patient centred outcomes. Patients with Coronavirus disease 2019 (COVID-19) were reported separately. Of the 3892/4546 patients without COVID-19, 2445 patients (62.8%) were from HDUs and 1447 (37.2%) from ICUs. Patients had a median age of 53 years (interquartile range [IQR] 38-68), with HDU patients being older but with a lower severity (APACHE II 6 [3-9] in HDUs vs 12 [7-17] in ICUs; p<0.001). One out of four patients were postoperative with 604 (63.4%) receiving emergency surgery. Readmission rate was 4.8%. Hypertension and diabetes were prevalent comorbidities, with a 4.0% HIV/AIDS rate. Invasive mechanical ventilation (IMV) was applied in 3.4% in HDUs vs. 47.6% in ICUs (P<0.001), with a duration of 7 days (IQR 3-21). There was a similar use of renal replacement therapy (4.0% vs. 4.7%; P<0.001). Vasopressor use was infrequent while half of patients received antibiotics. Average length of stay was 2 days (IQR 1-5). Crude HDU mortality rate was 6.5% in HDUs versus 30.5% in the ICUs (P<0.001). Of the 654 COVID-19 admissions, most were admitted in ICUs (72.3%) with a 33.2% mortality.

**Conclusions:**

We provide the first multicenter observational cohort study from an African ICU national registry. Distinct management features and outcomes characterise HDU from ICU patients.

**Study registration:**

Clinicaltrials.gov (reference number NCT05456217, date of registration 07 Nov 2022)

## Introduction

In synchrony with the increasing global burden of critically ill patients,^1^ critical care services in Kenya are rapidly evolving as an established healthcare service. The availability of both high dependency units (HDUs) and intensive care units (ICUs) is increasing in Kenya and other African countries.^2,3^ As of 2020, there were 54 critical care units in public, private and faith-based healthcare facilities spread out over 22 out of 47 counties in Kenya.^4,5^

**Understanding of demographics, management patterns and outcomes of patients accessing critical care units is pivotal for service evaluation and quality improvement, government resource planning and clinical trials sample size and equipoise assessments. This importance was accrued during the COVID 19 global pandemic.^8^ Few single centre studies assessed outcomes of patients in Kenyan ICUs, reporting high mortality and variable performance of existing prognostic score systems.**^6,7^ **A recent study landscaped the organisation, staffing and resources of both public and private critical care units in Kenya.**^8^ We still lack large multicenter studies describing patient epidemiological characteristics, patterns of management and patient centred outcomes.

Patients in Kenya access different types of critical care units with varying levels of structural organisation, organ support capabilities, resources and staffing patterns. While terminology varies, several classification systems exist to frame services provided by HDUs versus ICUs.^9^ Usually HDUs care for less sick patients, provide support for only one organ failure and have a lower nurse to patient ratio as compared to full feature ICUs.

To facilitate real-time patient level data collection enabling critical care service evaluation and identification of targets for quality improvement initiatives, the Kenyan Critical Care Registry was launched in December 2020 by the Critical Care Society of Kenya (CCSK) with the Collaboration for research, implementation and training in Critical Care in Asia and Africa (CCAA network) and Mahidol Oxford Research Unit (MORU).^10^ **The registry currently involves eleven units spread across six hospitals in five counties in Kenya. As other national ICU registries worldwide, it also provides benchmarking services, opportunity for service forecasting and allocation of resources.**^11–14^

In this multicenter registry based observational study, we sought to describe patient epidemiological characteristics, patterns of management and outcomes of critically ill patients referred to critical care units in Kenya. We also aimed to define variations in these features between HDU and ICU patients.

## Methods

### Study design and ethics approval

EPidemiology, clinical characteristics and Outcomes of adult admissions to high-dependency and intensive care units in Kenya’ (EPOK) was a multicentre registry-based prospective observational study. Data was collected from the Kenya Critical Care Registry housed under the Critical Care Society of Kenya (CCSK). Primary ethics approval for the registry deployment was sought from the Aga Khan University Institutional Ethics Review Committee (reference number 2019/IERC-89 approved on 26 November 2020) and for the present study (approval for ‘Baseline Kenya Critical Care Registry output’ with reference number 2021/IERC-125 on September 28th, 2021). The National Commission for science, technology and innovation (NACOSTI) licence was obtained (reference number 16058). Site approvals from NACOSTI-accredited ethical committees and/or administrative clearance were obtained from participating institutions prior to registry initiation. A waiver of informed consent was obtained. The study was registered on Clinicaltrials.gov (reference number NCT05456217). Preliminary results were previously reported in abstract form.^15^ The study is reported following the Strengthening of the reporting of Observational Studies in Epidemiology (STROBE) statement guidelines and checklists (Supplemental Table 1).^16^

### Participants

Consecutive patients of 18 years or older admitted to HDUs and ICUs from January 2021 to June 2022 within eleven Kenya Critical Care Registry network units were enrolled. Patient follow up lasted until HDU or ICU discharge.

### Study endpoints

**Descriptive endpoints included case-mix variables such as information on demographic characteristics like age, years, sex at birth, comorbidities, reasons for admission, source of admission and surgical status. The acute physiology and chronic health evaluation (APACHE) II score was used to describe severity of illness, with a range from 0 to 71**. Management features included organ support measures namely use of invasive mechanical ventilation, non invasive ventilation, high-flow nasal therapy (HFNT), use of vasoactive medication, sedatives and renal replacement therapy. Antibiotic prescriptions during the first 24h of patient care were also reported. **Patient-centred outcomes included duration of invasive mechanical ventilation, length of stay and crude mortality**.

### Registry structure

A preestablished critical care minimum dataset (Supplemental Table 2) was used in all centres participating in the study. Details of the registry structure, quality assurance and data safety are available in the supplementary material (page 2) and previously published work.^17^ The registry data flow is schematized in Supplemental Figure 1.

### Statistical Analysis Plan

For this descriptive study no formal sample size calculation was performed. All patients admitted to participating critical care units from the registry onset to June 2022 were included. The main grouping variable was the type of unit of admission (HDU versus ICU). Patients with a diagnosis of COVID-19 were described as a preestablished patient subgroup.

Continuous variables were presented as medians (IQR) and categorical variables as number and percentages. Continuous data were tested for normality using the Shapiro Wilk normality tests. Mann Witney and Chi-Square tests were used for comparisons of continuous and nominal data, respectively. The APACHE II score was calculated for all patients as originally described.^18^ **Missing values in the variables required for APACHE II score calculation were handled through imputation using the normal distribution method. For missing data in categorical variables we reported denominators and adjusted proportions accordingly.** The association between the grouping variable (admission to HDU or ICU) and mortality as a time–to–event was analysed with a Cox regression model, reporting the hazard ratio with 95%–confidence interval (CI). All analyses were performed using a two–sided superiority hypothesis test, with a significance level of 0.05. No corrections were performed for multiple comparisons across descriptive outcomes. Analyses were performed using software R (version 4.0.2, R Core Team, 2016, Vienna, Austria).

## Results

### Patient cohort and geographic distribution

**Figure 1:**
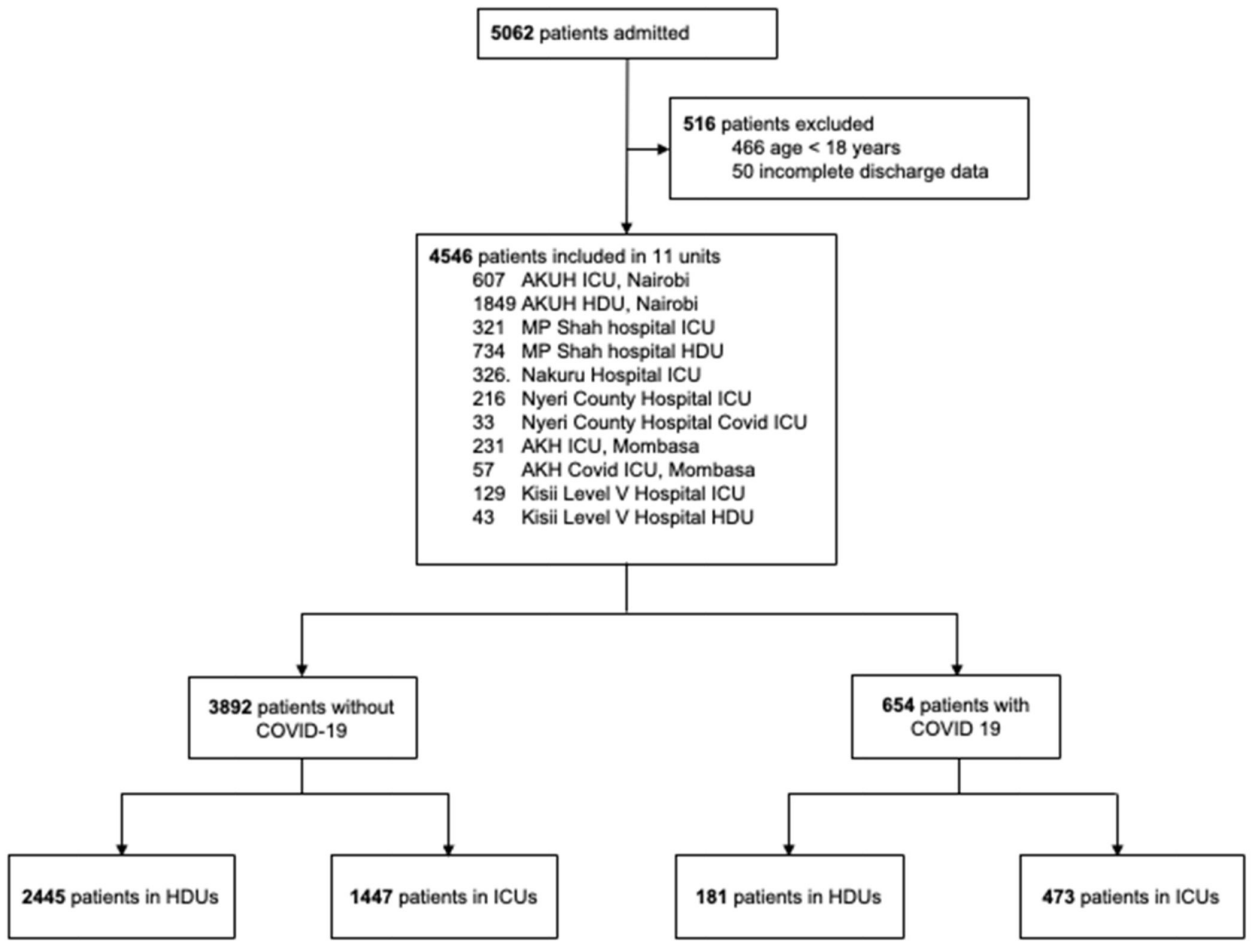
Patient flowchart

**Figure 2:**
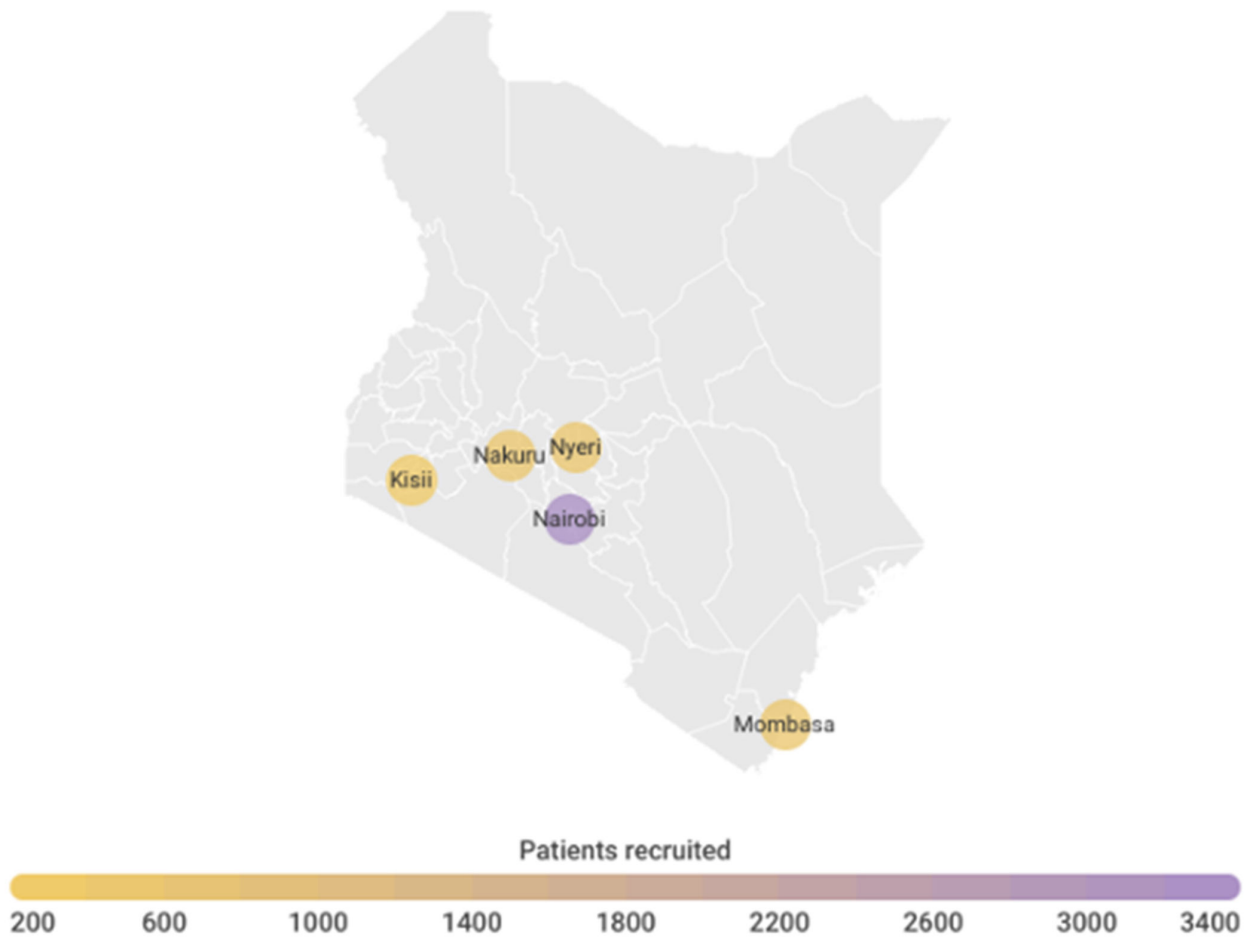
Geographical distribution of recruited patients in Kenya, stratified by county

A total of 4546 patients were recruited in the Kenya Critical Care registry from January 2021 to June 2022, with a rising number of monthly encounters in time (Supplemental Figure 2). Patient enrollment flowchart is shown in Figure 1. There was an imbalance in geographical distribution of patients with a wider representation of patients from the southern part of the country and the capital city Nairobi (Figure 2). The amount of missing data was low (Supplemental Table 2). Of the 3892 patients admitted for a reason different from SARS-CoV-2 infection, 2445 (62.8%) patients were admitted to HDUs and 1447 (37.2%) to ICUs. A total of 654 patients (14.4%) were confirmed positive for SARS-CoV-2 infection, with 27.6% being admitted to HDUs while 72.3% were ICU admissions.

### Units Characteristics

**A total of six mixed ICUs, two COVID ICUs and three HDUs participated in the study from six different hospitals. The units differed in terms of organisational, staffing and availability of resources** (Supplemental Table 3). **All units apart from one HDU had functional mechanical ventilators. A functional blood gas apparatus was missing in five units**.

### Case-mix and severity of illness

Demographic characteristics, reasons of admissions and reported comorbidities of patients admitted for reasons other than COVID-19, are summarised in Table 1. Median age was 53 (38-68) years, with HDUs admitting older patients (55 [40-70] years compared to ICUs (49 [34-64] years; p<0.001). Overall, 42% patients were female with a similar sex at birth distribution between the units. Main diagnostic categories were respiratory, neurological and cardiovascular. One out of four patients was admitted following a surgical operation with 63.4% being emergency procedures. Hypertension and diabetes mellitus were highly prevalent, with HIV prevalence reported at 4.0% (Supplemental Table 4). **Patients in HDUs had more frequent comorbidities as compared to ICUs**.

The severity of illness on admission measured by the APACHE II score was lower in HDUs (6 [3-9] vs 12 [7-17] in ICUs; p<0.001). Overall, 4.7% of patients were unconscious on admission, less frequently in HDUs (0.7% versus 11.6% in ICUs; p<0.001). Overall, blood gas analysis information was available in half of patients (49.8%), less frequently in ICU patients (Supplemental Table 5).

### Management features during first 24h

Only 3.4% of HDU patients were invasively ventilated versus 47.6% in the ICU (p<0.001; Table 2). **HFNT and non-invasive ventilation were seldom used in both HDUs and ICUs despite being available in half of the hospitals**. Sedation was used less frequently in HDUs as compared to ICUs (2.7% vs. 26.9%, P<0.001). Overall, vasopressor use rate in the first 24 hrs was 5.4% with a lower use in HDUs (2.7% versus 10.2% in ICUs; (p<0.001). RRT was used in 4.2% of patients, similarly in HDUs and ICUs. One out of two admitted patients received antimicrobial therapy during the first 24 hours of admission - less frequently in HDUs (P<0.001).

### Patient outcomes

**Figure 3:**
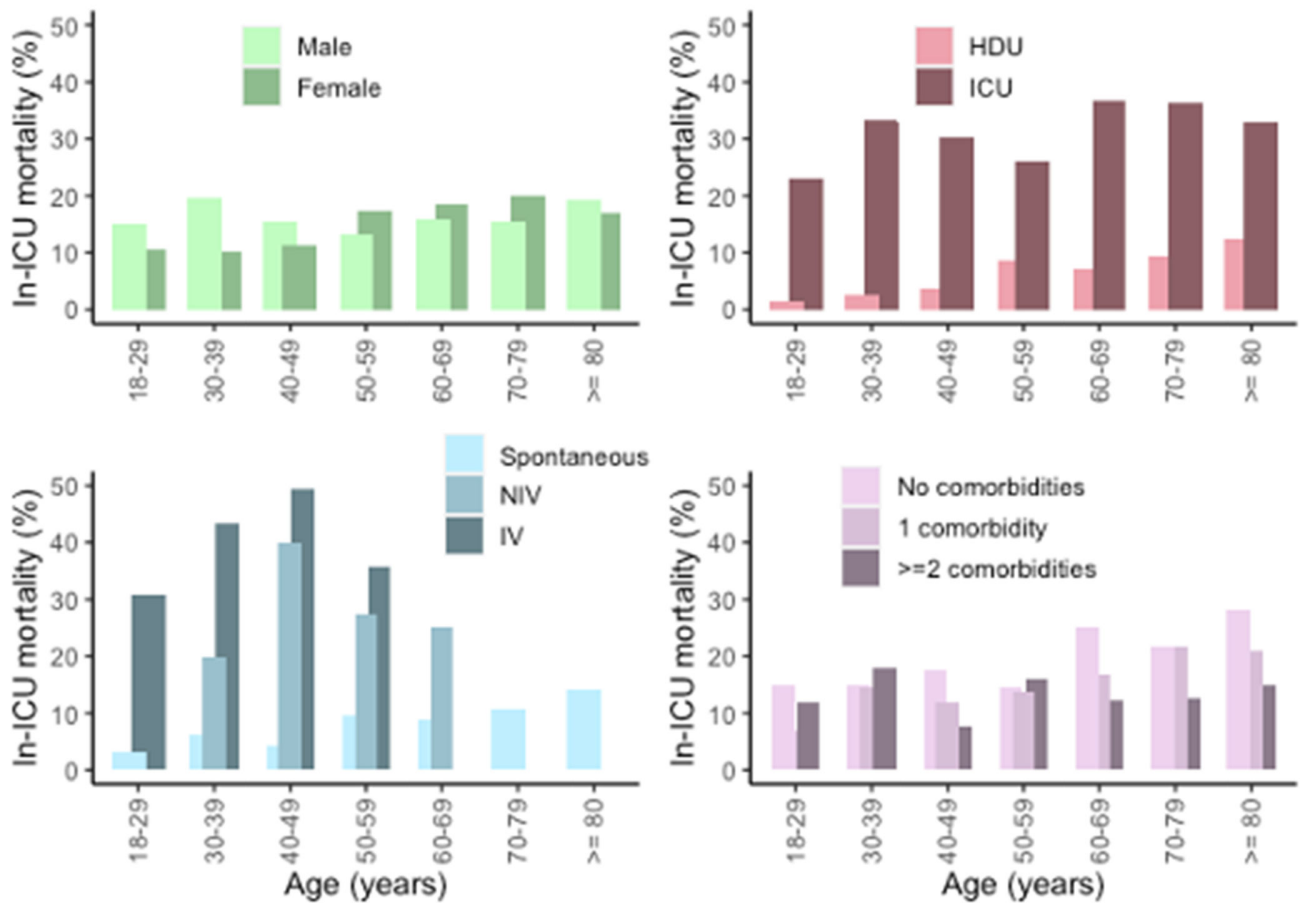
Crude ICU mortality stratified by age, sex, comorbidities, ICU/HDU admission status, and invasive mechanical ventilation for patients admitted to critical care units in Kenya (excluding COVID 19 patients) Data are from patients with a defined ICU discharge outcome; proportions of patients were calculated on the basis of complete case data for sex, comorbidities, ICU/HDU admission status, and invasive ventilation variables. ICU. intensive care unit; HDU, high dependency unit; HFNT, high flow nasal cannula, NIV, non invasive ventilation, IV, invasive ventilation

**Figure 4:**
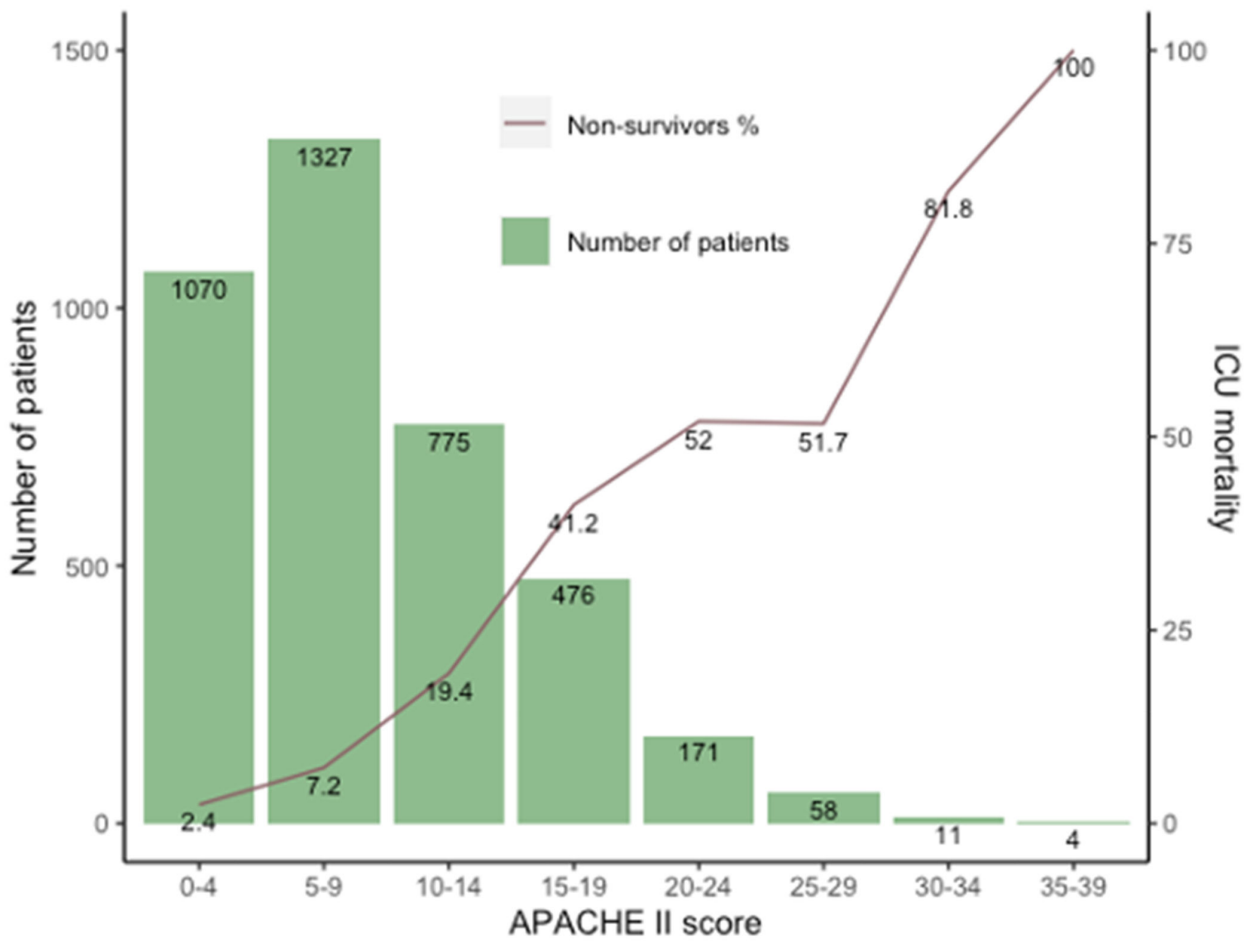
Distribution of Acute Physiology and Chronic Health Evaluation II scores and ICU mortality rates (excluding COVID-19 patients) APACHE, acute physiology and chronic health evaluation.

Patient outcomes are summarised in Table 2. Crude mortality stratified by age, sex, comorbidities, ICU/HDU admission status and invasive mechanical ventilation is shown in Figure 3. Mortality increased linearly with rising APACHE-II levels (Figure 4), with probability of survival in time being higher in HDUs compared to ICUs (HR for death in ICU compared to HDU 3.7 (95%CI 3.1-4.5; P<0.001). Duration of invasive mechanical ventilation for intubated patients was 7 days (3-21). Median length of stay in critical care was 2 days (1-5), being shorter in the HDU as compared to ICUs (2 [1-4] versus 3 [1-6] days; p<0.001). One out of twenty patients (5.3%) received a tracheostomy during ICU stay.

### COVID-19 patients subset

COVID-19 admissions were less frequent in HDUs as compared to ICUs. Peaks of admissions for COVID-19 were observed in March, July and November 2021. The median age was 62 years (IQR 49-72). Most admissions came from inpatient facilities (44.8%) and the emergency department (39%; Supplemental Table 6). The most common comorbidity was hypertension. Most patients were conscious (**77.7%;** Supplemental Table 7) and spontaneously breathing on admission (70.3%), with 14.7% of patients requiring invasive ventilation (2.8% in HDUs vs. 19.2% in ICUs, P<0.001). Use of sedatives, vasopressors and RRT was similar to patients without COVID-19 (Supplemental Table 8). Overall, 62.7% patients received antibiotics on admission (36.5% in HDUs vs 72.7% in ICUs, P<0.001). The overall mortality rate was 33% (21.0% in HDUs vs 37.8% in ICUs, P<0.001 - Supplemental Table 9).

## Discussion

This report describes demographic, clinical features and outcomes of a large cohort of patients admitted to critical care units in Kenya. This data is key in defining the context-specific epidemiology, forecasting the need of critical care resources, as well as informing hospital preparedness activities. These findings will also contribute to future planning of clinical trials in terms of equipoise establishment and sample size calculations.

**More than half of patients in the Kenyan registry were HDU admissions. This is a novel finding, highlighting the role of intermediate units in caring for critically ill patients with a low to medium degree of severity. While several research groups are attempting to establish minimum levels of critical care in Africa,**^19,20^ **exact guidelines, admission criteria and quality indicators for HDUs are still lacking. Registry based studies focusing on HDU patients characteristics were performed especially in Japan, focusing on pneumonia, COVID-19 and surgical patients.**^21,22^ **Single centre studies are available from African HDUs, but focused on organisational characteristics, cost-effectiveness and specific subpopulations such as obstetrics.**^2,3,23^ **EPOK’s findings pinpointing extensive clinical and outcome features, provide the first patient-level multicenter data specifically assessing this group of patients against the group admitted to ICUs**.

Our findings on case-mix and epidemiological characteristics are in line with previous reports from Kenya and other African countries.^6,7^ The age of critically ill patients in Kenya was relatively low, with overall half of patients being between 38 and 68 years of age, with even younger patients accessing ICUs. Of note, we could not show a clear linear increase of mortality with age. Six out of ten patients had one or more comorbidities with a spectrum of chronic conditions including hypertension, diabetes, chronic kidney disease and neoplastic disorders. A quarter of patients arrived in the critical care unit after surgery, in line with other international observational studies,^24–27^ although less than reported in Uganda^28^ and Malawi.^29^ In fact, critical care registries such as the one deployed for this study were deemed important to tackle the high perioperative mortality in Africa.^30^

**The findings on organ support in patients accessing Kenyan HDUs and ICUs present some peculiarities. There was a comparable use of non-invasive mechanical ventilation in HDUs as ICUs, while invasive mechanical ventilation clearly remains an ICU feature**. **Of note, the use of renal replacement therapy was equal in HDUs compared to ICUs, emphasising the important role that intermediate level units play in renal organ support**. Patients in ICUs received similar rates of mechanical ventilation and RRT as compared to another registry-based cohort in Brazil, but consistently less cardiovascular support.^11^ The rate of cardiovascular support was also lower compared to cohorts from India^13^, and from previous studies in Kenya.^6,7^ Whether this represents underreporting or actual underuse of cardiovascular support remains to be elucidated. With regards to respiratory support, we observed that HFNT is still rarely used in both HDUs and ICUs, despite efforts of increasing non invasive ventilation support occurring during the pandemic. Whether patients not receiving invasive ventilation should be admitted to ICUs is a debated issue in other national registries^31^. Scaling up of HFNT use or zooming in on the use of cardiovascular support represent potential targets of quality improvement initiatives focusing on adoption and fidelity of interventions.

Outcomes of critically ill patients in our cohort seem to show an improving trend compared to past reports. A previous single centre report in 2018 including 450 ICU patients reported a mortality above 50%,^7^ while in our study it is just above 30% for the ICU cohort. The 2018 study included patients that were more frequently ventilated or received other organ support measures, limiting the meaningfulness of direct comparisons.^7^ EPOK’s ICU mortality is comparable to a more recent study looking at outcomes of one single well resourced ICU in Kenya.^6^ However, the observed ICU mortality exceeding 30% in a mixed ICU population is higher than previously found in a large multicenter point prevalence study in India that had comparable APACHE II scores on admission^24^ or from registry based evaluations from Brazil^11^ and India.^13^ A recent analysis focusing on mechanically ventilated patients in Ethiopia reported an ICU mortality of 49%, identifying as relevant risk factors being diabetic, having GSC < 8, and nighttime admissions^32^. This is considerably higher than what was observed in middle income countries in Asia.^25^ Subsequent analysis will be needed to define modifiable factors for mortality in Kenya, both in ventilated and non ventilated subgroups.

The proportion of COVID-19 admissions (14%) was comparable to other countries during the pandemic period. The SARS-CoV2 vaccination program was initiated in March 2021 and there was a large unvaccinated vulnerable geriatric population at the beginning of the EPOK study. SARS- CoV2 admissions were mainly internal referrals, or from hospitals without critical care services. Patients’ age and comorbidities were in line with findings from the African COVID-19 Critical Care Outcomes Study (ACCCOS) cohort.^33^ Length of stay was also similar around a median of 6-7 days. Crude mortality is difficult to compare as ACCCOS reported 30 days mortality (48%) while EPOK reports death at ICU discharge. **A multinational registry-based study concluded that the pandemic particularly affected outcomes of non-COVID patients in LMICs, but we could not corroborate this hypothesis in our data.**^34^

The EPOK study has several limitations. Firstly, only one fifth of critical care units in Kenya was represented in the study, although the mix allowed for both private and public centres with a geographic distribution that mirrors the one found in a recent landscaping ICU survey.^8^ **Secondly, willingness of participating units to join a national registry could have led to selection bias towards inclusion of units with better resources or outcomes. The HDU cohort was unbalanced towards one high-resourced HDU in Nairobi.** Similar to other registry-based studies, access to patient’ data was restricted to data collectors and site leads, and no formal source data verification was performed. **In addition, we lacked data on several key domains such as entry criteria in the units, complications occurring during ICU stay, granular data on ventilation and hemodynamic variables, long term mortality as well as institutional policies on end of life and palliative care. The study was performed amid COVID-19 surges, thus admission patterns in the non-COVID units may have differed from the norm.**

## Conclusion

EPOK’s findings extend our knowledge on demographics, clinical features and short term outcomes of patients being admitted to critical care units in Kenya. The defined timeframe within which data was gathered limited the effect of practice changes over time, while allowing to account for seasonal case-mix variations and inclusion of the COVID-19 patient subgroup. This data establishes a basis for sample size calculations for future critical care care trials in Kenya, provides context specific information on quality improvement targets and contemporary service evaluation from a representative sample of public and private units in the country.

## Supplementary Material

Supplemental Data File

## Figures and Tables

**Table 1:** Demographic characteristics, severity level, reason and sources of admission of patients (excluding COVID-19 patients).

Variables	All patients (n=3892)	Patients in HDU (n=2445)	Patients in ICU (n=1447)	P-value
**Demographics**				
Age, years	53 (38-68)	55 (40-70)	49 (34-64)	0.000*
Sex, female	1633 (42.0)	1058 (43.3)	575 (39.7)	0.071
**APACHE II score**	8 (4-13)	6 (3-9)	12 (7-17)	0.000*
**Reason of admission to ICU** *				0.000*
Cardiovascular	1145 (29.4)	704 (28.8)	441 (30.5)	
Neurologic	1149 (29.5)	618 (25.3)	531 (36.7)	
Respiratory	837 (21.5)	440 (18)	397 (27.4)	
Gastrointestinal	827 (21.2)	508 (20.8)	319 (22)	
Genitourinary	463(11.9)	225 (9.2)	238 (16.4)	
Metabolic/Endocrine	349 (9)	246 (10.1)	103 (7.1)	
Trauma	335 (8.6)	107 (4.4)	228 (15.8)	
Haematology	150 (3.9)	104 (4.3)	46 (3.2)	
Musculoskeletal/Skin	146 (3.8)	77 (3.1)	69 (4.8)	
Transplant	8 (0.2)	5 (0.2)	3 (0.2)	
**ICU admission source**				0.000*
**Same Hospital**				
ED	2028/3862 (52.5)	1501/2420(62.0)	527/1442 (36.5)	
Ward	848/3862 (22.0)	474/2420 (19.6)	374/1442 (25.9)	
Operating theatre	550/3862 (14.2)	222/2420 (9.2)	328/1442 (22.7)	
ICU/HDU	259/3862 (6.7)	181/2420(7.5)	78/1442 (5.4)	
**Other Hospital**				
ED	39/3862 (1.0)	5/2420 (0.2)	34/1442 (2.4)	
Ward	69/3862 (1.8)	4/2420 (0.2)	65/1442 (4.5)	
Operating theatre	13/3862 (0.3)	0/2420 (0.0)	13/1442 (0.9)	
ICU/ HDU	25/3862 (0.6)	9/2420 (0.4)	16/1442 (1.1)	
Operating theatre, unclassified	31/3862 (0.8)	24/2420 (1.0)	7/1442 (0.5)	
**Readmissions**	185/3892 (4.8)	123/2445 (5.0)	62/1447 (4.3)	0.314
**Admission type**				
Operative	952/3892 (24.5)	495/2445 (20.2)	457/1447 (31.6)	0.000*
Non-operative	2940/3892 (75.5)	1950/2445 (79.8)	990/1447 (68.4)	
**Emergency surgery for operative**	604/952 (63.4)	286/495 (57.8)	318/457 (69.6)	0.000*

Data is presented as frequency (%) or median (interquartile range)

* classification based on APACHE IV coding. HDU, high dependency unit; ICU, intensive care unit; ED, emergency department.

**Table 2:** Management features and patient outcomes (excluding COVID-19 patients)

Variables	All patients (n=3892)	Patients in HDU (n=2445)	Patients in ICU (n=1447)	P-value
**MANAGEMENT**				
**Ventilation status on admission**				0.000*
Spontaneous	3047/3869 (78.8)	2332/2444 (95.4)	715/1425 (50.2)	
HFNT	3/3869 (0.1)	0/2444 (0)	3/1425 (0.2)	
NIV	48/3869 (1.2)	30/2444 (1.2)	18/1425 (1.3)	
Invasive ventilation	771/3869 (19.9)	82/2444 (3.4)	689/1425 (48.4)	
**Therapeutics**				
Use of sedatives	423/3889 (10.9)	34/2444 (1.4)	389/1425 (27.3)	0.000*
Use of vasopressors	210/3889 (5.4)	65/2444 (2.7)	145/1425 (10.2)	0.000*
Renal replacement therapy	165/3889 (4.2)	97/2444 (4.0)	68/1425 (4.8)	0.267
Antimicrobial use	1936/3889 (49.7)	941/2444 (38.5)	995/1425 (69.8)	0.000*
**OUTCOMES**				
**Mortality at ICU discharge**	601/3892 (15.4)	160/2445 (6.5)	441/1447(30.5)	0.000*
**Discharge destination for survivors**				
Ward	2682/3291 (81.5)	1956/2285 (85.6)	726/1006 (72.2)	0.000*
ICU	128/3291 (3.9)	92/2285 (4.0)	36/1006 (3.6)	
Home	211/3291 (6.4)	186/2285 (8.1)	25/1006 (2.5)	
HDU	151/3291 (4.6)	3/2285 (0.1)	148/1006 (14.7)	
Other hospitals	55/3291 (1.7)	21/2285 (0.9)	34/1006 (3.4)	
Transfer for specialist care	13/3291 (0.4)	5/2285 (0.2)	8/1006 (0.8)	
Other reasons	51/3291 (1.5)	22/2285 (1.0)	29/1006 (2.9)	
Left against medical advice*	20/3291 (0.6)	14/2285 (0.6)	6/1006 (0.4)	0.000*
Discharge upon patient request**	39/3291 (1.2)	15/2285 (0.7)	24/1006 (2.4)	0.000*
**Other outcomes**				
Length of stay, days	2 (1-5)	2 (1-4)	3 (1-6)	0.000*
Duration of MV, days (n=606)	8 (3-21)	NA#	8 (3-21)	NA#
Tracheostomy	32/606 (5.3)	NA#	32/606 (5.3)	NA#

Data is presented as frequency (%) or median (interquartile range) Abbreviations: ICU, intensive care unit; HDU, high dependency unit; HFNT, high flow nasal cannula; NIV, non invasive ventilation; MV, mechanical ventilation.

* defined as the patient leaving the ICU against the advice of their medical team.

** defined as the decision to discharge from ICU made by a patient or family and facilitated by the clinical team (e.g. transfer to another hospital at patient’s request).

# daily registry data not available in HDU

## Data Availability

The datasets used and analysed during the current study are available on motivated request. For further information and access to the data, please contact the Critical Care Asia Africa (CCAA) data access committee (DAC@nicslk.com) and quote the manuscript, your institution and provide return correspondence information.

## Key Points

### Question

What are the clinical characteristics and outcomes of critically ill patients admitted to critical care units in Kenya?

### Findings

We leveraged the novel Kenya Critical Care Registry which uses a cloud based platform and common data model to report contemporary data on more than four thousand admissions to high dependency units (HDUs) and intensive care units (ICUs) in the country.

### Meanings

EPOK’s large dataset is key in defining the context-specific epidemiology and outcomes. This information is pivotal for clinical trials equipoise assessment, sample size estimations and national critical care resources forecasting.
