# Supplementary material for: EPidemiology, clinical characteristics and Outcomes of 4546 adult admissions to high-dependency and intensive care units in Kenya (EPOK): a multicentre registry-based observational study
Source: Crit Care Explor. Author manuscript; Available in PMC 2024 Feb 14. (PMC7615640; doi:10.1097/CCE.0000000000001036)
Supplement: Supplemental Data File [file EMS192902-supplement-Supplemental_Data_File.pdf]

SUPPLEMENTARY MATERIAL

EPidemiology, clinical characteristics and Outcomes of 4546 adult admissions to high-dependency and intensive care units in Kenya (EPOK): a multicentre registry-based observational study

Contents:

Additional details on Kenya Critical Care Registry data collection procedures 2

Supplemental Figure 1. Data flow, data access and output examples in the Kenya Critical Care Registry 3

Supplemental Figure 2. Number of encounters in the Kenya Critical Care registry per month from January 2021 to June 2022 4

Supplemental Table 1. STROBE checklist 5

Supplemental Table 2. Critical Care Registry Minimum Dataset (CCMDS) and data availability between January 2021 and June 2022 7

Supplemental Table 3: Characteristics of participating units 8

Supplemental Table 4. Comorbidities of patients (excluding COVID-19 patients) 9

Supplement Table 5: Vital signs on admission and availability of blood gas analysis variables (excluding COVID-19 patients) 10

Supplement Table 6. Clinical characteristics of the COVID-19 patient subgroup 11

Supplement Table 7: Clinical condition on admission and main laboratory values for the COVID-19 subgroup. 13

Supplement Table 8: Management characteristics for the COVID-19 subgroup 14

Supplement Table 9. Patients outcomes for the COVID-19 subgroup 15

Additional details on Kenya Critical Care Registry data collection procedures

Once entered electronically on password-protected tablets or computers at participating facilities, the data is automatically encrypted before it leaves the institution for the visualization loop within a ring-fenced server at (Nat Intensive Care - Mahidol Oxford Research Unit) NICS-MORU, where it is unencrypted and aggregated for automated visualization (**Supplemental Figure 1**). The processed data is then re-encrypted before it is transferred back to the national server, where it is automatically unencrypted and available for review by authorized personnel, through a secure two step login procedure. An audit trail is created any time the registry is accessed, in order to record user logins, retrieval or modifications of data. The data processing stage is fully automated and patient-level data is not made accessible outside of participating facilities. The team of investigators are only able to view de-identified aggregate data whose source institution is not identifiable. As in other ICU registries, identifiable patient data was restricted to authorized hospital staff. Data extraction and analysis only concerned de-identified data. The NICS-MORU team has access to the system only for purposes of system support and maintenance.

Supplemental Figure 1. Data flow, data access and output examples in the Kenya Critical Care Registry

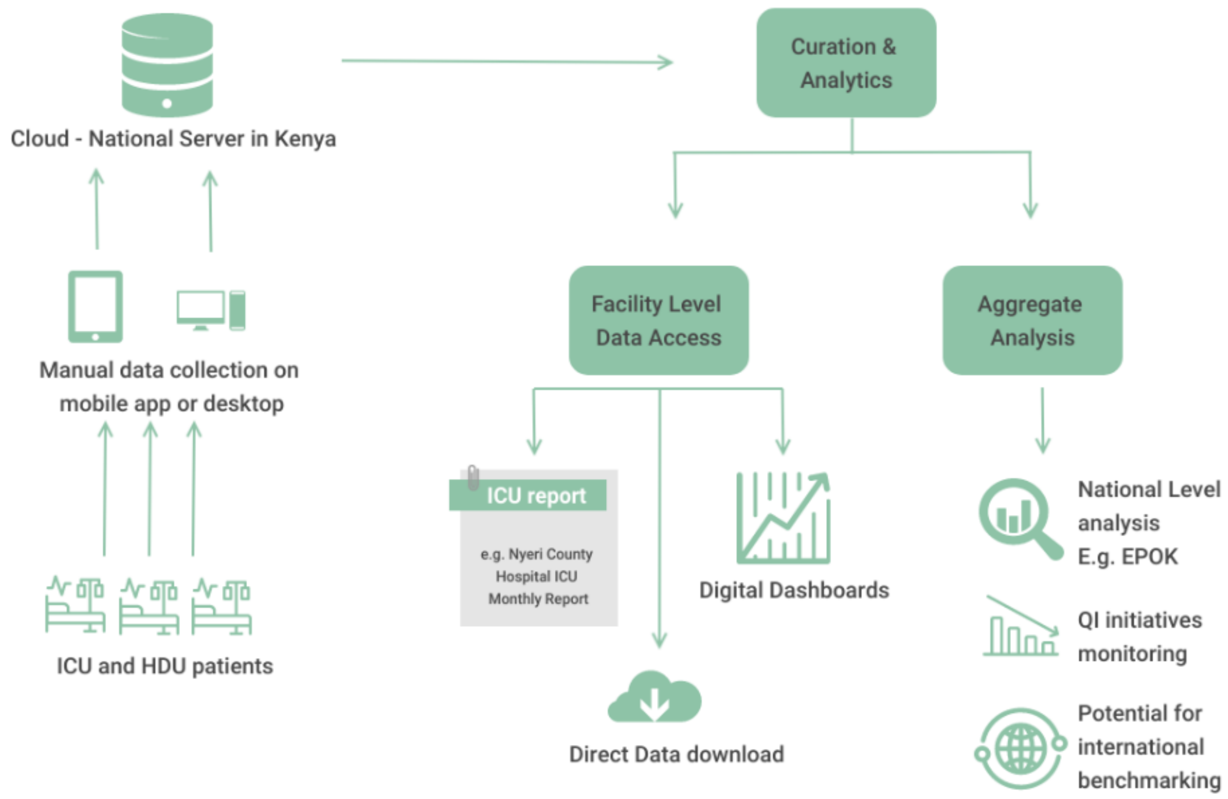

Supplemental Figure 2. Number of encounters in the Kenya Critical Care registry per month from January 2021 to June 2022
SARI, severe acute respiratory infections i.e. patients with SARS-CoV2 diagnosis on admission.

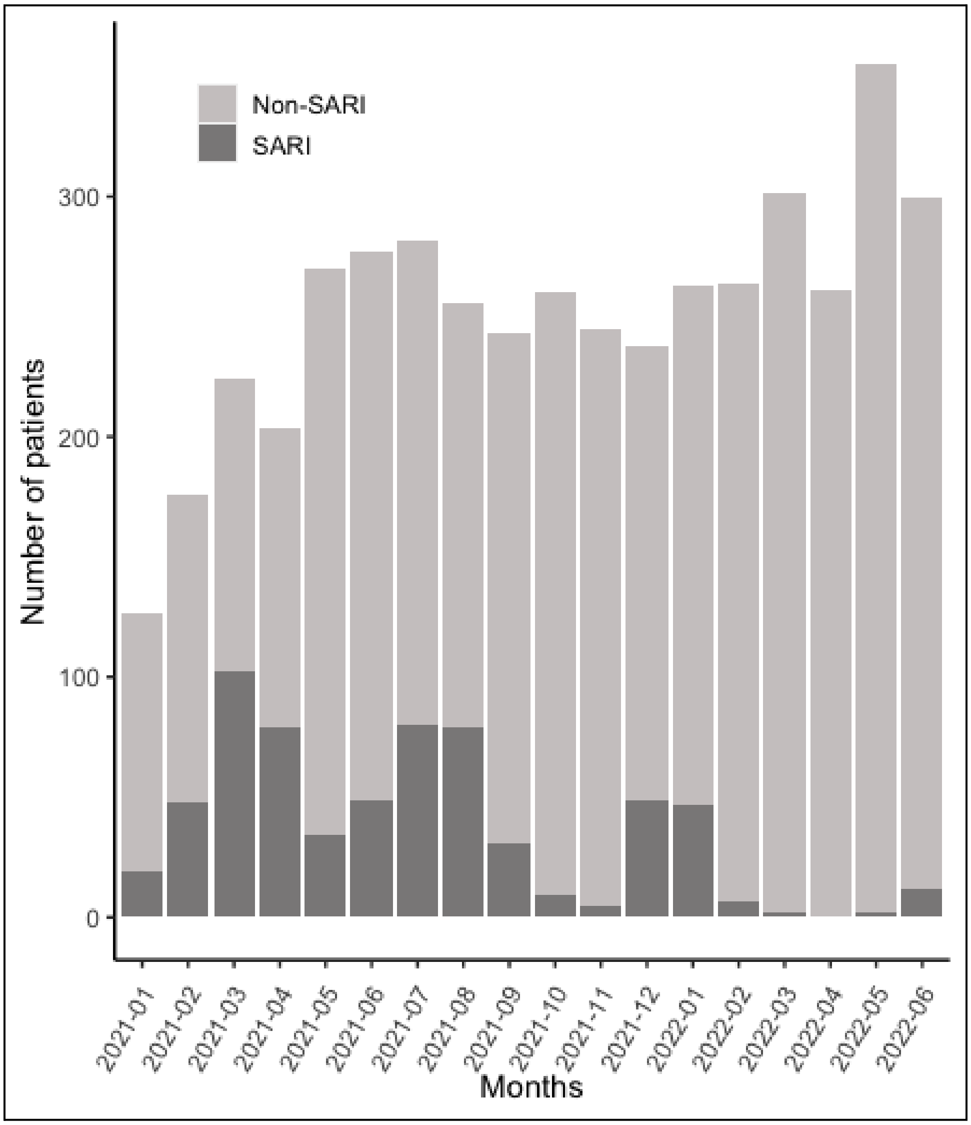

Supplemental Table 1. STROBE checklist

	Item No	Recommendation	Manuscript section
Title and abstract	1	(*a*) Indicate the study's design with a commonly used term in the title or the abstract	Methods
		(*b*) Provide in the abstract an informative and balanced summary of what was done and what was found	Abstract
**Introduction**			
Background	2	Explain the scientific background and rationale for the investigation being reported	Introduction
Objectives	3	State specific objectives, including any prespecified hypotheses	Introduction
**Methods**			
Study design	4	Present key elements of study design early in the paper	Methods
Setting	5	Describe the setting, locations, and relevant dates, including periods of recruitment, exposure, follow-up, and data collection	Methods
Participants	6	(*a*) Give the eligibility criteria, and the sources and methods of selection of participants	Methods
Variables	7	Clearly define all outcomes, exposures, predictors, potential confounders, and effect modifiers. Give diagnostic criteria, if applicable	Methods
Data sources/ measurement	8*	For each variable of interest, give sources of data and details of methods of assessment (measurement). Describe comparability of assessment methods if there is more than one group	Methods and supplement
Bias	9	Describe any efforts to address potential sources of bias	Methods
Study size	10	Explain how the study size was arrived at	Methods
Quantitative variables	11	Explain how quantitative variables were handled in the analyses. If applicable, describe which groupings were chosen and why	Methods
Statistical methods	12	(*a*) Describe all statistical methods, including those used to control for confounding	Methods
		(*b*) Describe any methods used to examine subgroups and interactions	Methods
		(*c*) Explain how missing data were addressed	Methods
		(*d*) If applicable, describe analytical methods taking account of sampling strategy	Not applicable
		(*e*) Describe any sensitivity analyses	Not applicable
**Results**			
Participants	13*	(a) Report numbers of individuals at each stage of study—eg numbers potentially eligible, examined for eligibility, confirmed eligible, included in the study, completing follow-up, and analysed	Results
		(b) Give reasons for non-participation at each stage	Not applicable
		(c) Consider use of a flow diagram	Figures
Descriptive data	14*	(a) Give characteristics of study participants (eg demographic, clinical, social) and information on exposures and potential confounders	Results
		(b) Indicate number of participants with missing data for each variable of interest	Results and Supplement
Outcome data	15*	Report numbers of outcome events or summary measures	Results
Main results	16	(*a*) Give unadjusted estimates and, if applicable, confounder-adjusted estimates and their precision (eg, 95% confidence interval). Make clear which confounders were adjusted for and why they were included	Not applicable
		(*b*) Report category boundaries when continuous variables were categorized	Not applicable

		(*c*) If relevant, consider translating estimates of relative risk into absolute risk for a meaningful time period	Not applicable
Other analyses	17	Report other analyses done—eg analyses of subgroups and interactions, and sensitivity analyses	Results and Supplement
**Discussion**			
Key results	18	Summarise key results with reference to study objectives	Results
Limitations	19	Discuss limitations of the study, taking into account sources of potential bias or imprecision. Discuss both direction and magnitude of any potential bias	Discussion
Interpretation	20	Give a cautious overall interpretation of results considering objectives, limitations, multiplicity of analyses, results from similar studies, and other relevant evidence	Discussion
Generalisability	21	Discuss the generalisability (external validity) of the study results	Discussion
**Other information**			
Funding	22	Give the source of funding and the role of the funders for the present study and, if applicable, for the original study on which the present article is based	Funding section

Supplemental Table 2. Critical Care Registry Minimum Dataset (CCMDS) and data availability between January 2021 and June 2022

Field name	Availability N (%) N=3892	Field name	Availability N(%) N=3892
Patient's name	3892 (100.0)	Respiratory rate	3869 (99.4)
Medical record number	3892 (100.0)	Heart rate	3869 (99.4)
Age	3892 (100.0)	Temperature	3869 (99.4)
Sex	3892 (100.0)	Glasgow coma scale (eye)*	3867 (99.4)
Date of hospital admission	3891 (100.0)	Glasgow coma scale (verbal)*	3867 (99.4)
Time of hospital admission*	3892 (100.0)	Glasgow coma scale (motor)*	3867 (99.4)
Date of ICU admission	3892 (100.0)	AVPU condition	3654 (93.9)
Time of ICU admission*		Blood glucose*	3558 (91.4)
ICU admission source	3862 (99.2)	Pain score*	3767 (96.8)
Readmission	3892 (100)	Haemoglobin	3540 (91.0)
Date of previous discharge*	3747 (96.3)	Platelet	3524 (90.5)
Type of admission	3892 (100.0)	Packed cell volume	2312 (59.4)
Emergency Surgery	3892 (100.0)	White blood cell count	3522 (90.5)
Reason for admission (operative)	3892 (100.0)	Serum Sodium	3465 (89.0)
Reason for admission (disorder)	3892 (100.0)	Serum Potassium	3465 (89.0)
Reason for admission (disorder)	3892 (100.0)	Serum HCO3	2265 (58.2)
SARI diagnosis	3892 (100.0)	Serum Creatinine	3370 (86.6)
Date of earliest symptoms*	0 (0.0)	Serum bilirubin	1794 (46.1)
Comorbidities	3892 (100.0)	Blood urea	3400 (87.4)
Ventilation	3869 (99.4)		
Route of Mechanical ventilation	3869 (99.4)	Date of discharge	3892 (100)
High flow oxygen	3869 (99.4)	Time of discharge	
FiO_2	3388 (87.1)	Discharge status	3892 (100)
PaO_2	1931 (49.6)	Discharge destination	3892 (100)
Arterial pH	1941 (49.9)	CPR status*	3892 (100)
Sedated	3869 (99.4)	SARI diagnosis on discharge	3608 (92.7)
Cardiovascular support	3869 (99.4)	SARI, yes	3608 (92.7)
Vasoactive therapy	3869 (99.4)	Type of test	3608 (92.7)
Renal replacement therapy	3869 (99.4)	Withdrawal of treatment*	3892 (100)
Antimicrobial use	3869 (99.4)	Left against medical advice	3291 (84.6)
Antimicrobial type	3869 (99.4)	Discharge upon patient request	3274 (84.1)
Systolic blood pressure	3868 (99.4)	Date of hospital discharge*	2484 (63.8)
Diastolic blood pressure	3868 (99.4)	Hospital discharge status*	2549 (65.5)

*not reported in current analysis

Supplemental Table 3: Characteristics of participating units

	AKH COVID ICU, Mombasa	AKH Mixed ICU, Mombasa	Mp Shah ICU, Nairobi	MP Shah HDU, Nairobi	AKUH Mixed ICU, Nairobi	AKUH HDU, Nairobi	Nakuru hospital Mixed ICU	Nyeri County Referral Hospital COVID ICU	Nyeri County Referral Hospital Mixed ICU	Kisii Teaching and Referral hospital ICU	Kisii Teaching and Referral hospital HDU
**ORGANIZATION**											
Hospital status	PNFP	PNFP	PNFP	PNFP	PNFP	PNFP	Public	Public	Public	Public	Public
Number of beds in Hospital	143	143	250	250	258	258	1000	250	250	650	650
Number of beds in unit	20	4	7	9	11	16	9	3	6	6	3
Yearly admissions	48	123	180	NA	500	1500	300	38	165	122	27
Model of care	Open	Open	Open	Open	Open	Open	Open	Closed	Closed	Open	Open
**STAFFING**											
Specialty of in-charge	Ane	Ane	Ane	Ane	Med	Med	Ane	Ane	Ane	Ane	Ane
Availability of dedicated clinician	NC	NC	C	C	NC	NC	No	No	No	NC	No
Number of nurses in the unit	12	13	36	24	46	44	30	11	15	29	0
Nurse to bed ratio day time	1:2	1:2	1:1	1:2	1:1	1:2	1:2	1:1	1:3	1:2	1:2
Nutritionist	On consult	On consult	On consult	On consult	On consult	On consult	Dedicated	On consult	On consult	Dedicated	On consult
Physiotherapist	On consult	On consult	On consult	On consult	On consult	On consult	Dedicated	On consult	On consult	Dedicated	On consult
Counsellor or Psychologist	On consult	On consult	On consult	On consult	On consult	On consult	On consult	On consult	On consult	No	no
Pharmacist	On consult	On consult	On consult	On consult	Dedicated	On consult	On consult	On consult	On consult	On consult	On consult
**RESOURCES**											
Isolation rooms in the unit	>3	none	2	9	11	4	1	3	1	1	0
Functional Mechanical Ventilators	2	5	8	2	11	3	20	5	14	12	0
Functional HFNT machines	2	3	5	2	5	2	0	1	0	0	0
BGA machine available	Yes	Yes	Yes	Yes	Yes	Yes	Yes	Yes	Yes	Yes	No
Functional BGA machine	Yes	Yes	Yes	Yes	Yes	Yes	No	No	No	No	No
Beds with pressure relieving mattress	2	4	7	NA	11	16	6	3	6	4	NA
Isolation rooms	>3	0	2	NA	11	4	1	3	1	1	NA

AKH, Aga Khan Hospital; AKUH, Aga Khan University Hospital; NC, non consultant doctor; C, consultant; CCN, critical care nursing; Ane, specialist in anesthesiology or intensive care medicine; Med, specialist in internal medicine or similar discipline; BGA, blood gas analysis.

Supplemental Table 4. Comorbidities of patients (excluding COVID-19 patients)

Variables	All patients (n=3892)	Patients in HDU (n=2445)	Patients in ICU (n=1447)	P-value
**Comorbidities top 15**				
Hypertension	1383 (35.6)	985 (40.3)	398 (27.5)	0.000*
Type 2 diabetes	398 (10.5)	270 (11.0)	128 (8.8)	0.016*
Type 1 diabetes	335 (8.5)	251 (10.3)	84 (5.8)	0.000*
Cardiovascular diseases	144 (3.7)	106 (4.3)	38 (2.6)	0.006*
Hypothyroidism	136 (3.5)	108 (4.4)	28 (1.9)	0.000*
Dyslipidemia	137 (3.4)	103 (4.2)	34 (2.3)	0.002*
Diabetes unclassified	134 (3.4)	113 (4.6)	21 (1.5)	0.000*
HIV/AIDS	156 (4.0)	86 (3.5)	70 (4.9)	0000*
Renal failure - moderate to severe	108 (2.8)	81(3.3)	27(1.9)	0.008*
Renal failure requiring dialysis	84 (2.2)	58 (2.4)	26 (1.8)	0.232
Asthma	81 (2.1)	54 (2.2)	27 (1.9)	0.469
Metastatic cancer	64 (1.6)	53 (2.2)	11 (0.8)	0.0008*
Cancer	51 (1.3)	36 (1.5)	15 (1.0)	0.248
Rheumatological condition	49 (1.2)	43 (1.8)	6 (0.4)	0.002*

Data is presented as frequency (%) or median (interquartile range)
*classification based on APACHE IV coding.
HDU, high dependency unit; ICU, intensive care unit; ED, emergency department;

Supplement Table 5: Vital signs on admission and availability of blood gas analysis variables (excluding COVID-19 patients)

Variables	All patients (n=3892)	Patients in HDU (n=2445)	Patients in ICU (n=1447)	P-value
**Vital signs on admission**				
Systolic blood pressure	n=3868<br>123 (108-140)	n=2444<br>123 (110-139)	n=1424<br>121(104-140)	0.005*
Diastolic blood pressure	n=3868<br>72 (62-84)	n=2444<br>72 (62-82)	n=1424<br>73 (61-86)	0.071
Respiratory rate	n=3869<br>20 (17-22)	n=2444<br>20 (17-22)	n=1425<br>20 (17-24)	0.000*
Heart rate	n=3869<br>89 (76-106)	n=2444<br>86 (75-100)	n=1425<br>96 (79-114)	0.000*
Temperature	n=3869<br>36.6 (36.5-36.8)	n=2444<br>36.6 (36.5-36.8)	n=1425<br>36.6 (36.2-36.9)	0.003*
**Glasgow coma scale**	n=3867<br>15 (11-15)	n=2444<br>15 (15-15)	n=1423<br>10 (3-15)	0.000*
**AVPU condition** (%)	n=3732	n=2356	n=1376	0.000*
Alert	2835 (76.0)	2230 (94.7)	605 (44.0)	
Verbal	238 (6.4)	62 (2.6)	176 (12.8)	
Pain	156 (4.2)	11 (0.5)	145 (10.5)	
Unresponsive	184 (4.9)	16 (0.7)	168 (12.2)	
Sedated	319 (8.5)	37 (1.6)	282 (20.5)	
**FiO_2**	0.3 (0.2-0.5)	0.2 (0.2-0.3)	0.5 (0.3-0.7)	0.000*
**Blood gas analysis**	n=1941	n=1422	n=519	
PaO_2, mmHg	76 (65-92)	75 (64-87)	83 (69-126)	0.000*
Arterial pH	7.4 (7.3-7.4)	7.4 (7.3-7.4)	7.4 (7.3-7.4)	0.000*

Data is reported as median (interquartile range) or as n(%).
Abbreviations: HDU, high dependency unit; ICU, intensive care unit; FiO_2, fraction of inspired oxygen: PaO_2, oxygen tension in arterial blood;

Supplement Table 6. Clinical characteristics of the COVID-19 patient subgroup

Variables	All patients (n=654)	Patients in HDU (n=181)	Patients in ICU (n=473)	P-value
**Demographics**				
Age, years	n=654<br>60 (49-72)	n=181<br>62 (49-74)	n=473<br>59 (48-71)	0.118
Female	223/654 (34.1)	73/181 (40.3)	150/473 (31.7)	0.047*
**Reason of admission to ICU***	n=654	n=181	n=473	
**Non-operative**				
Cardiovascular	60/654(9.2)	17/181(9.4)	43/473(9.1)	0.293
Neurologic	22/654(3.4)	9/181(5.0)	13/473(2.7)	
Respiratory	607/654 (92.8)	161/181(89.0)	446/473(94.3)	
Gastrointestinal	11/654(1.7)	4/181(2.2)	7/473(1.5)	
Genitourinary	13/654 (2.0)	7/181(3.9)	6/473(1.3)	
Metabolic/Endocrine	36/654 (5.5)	10/181(5.5)	26/473(5.5)	
Trauma	4/654 (0.6)	0/181(0.0)	4/473(0.8)	
Hematology	6/654 (0.9)	1/181(0.6)	5/473(1.1)	
Musculoskeletal/Skin	2/654 (0.3)	1/181(0.6)	1/473(0.2)	
**Operative**				
Cardiovascular	3/654 (0.5)	1/181(0.6)	1/473(0.2)	
Neurologic	5/654(0.8)	1/181(0.6)	4/473(0.8)	
Respiratory	0/654(0)	0/181(0)	0/473(0)	
Gastrointestinal	3/654(0.5)	0/181(0)	3/473(0.6)	
Genitourinary	3/654(0.5)	0/181(0)	3/473(0.6)	
Metabolic/Endocrine	0/654(0)	0/181(0)	0/473(0)	
Trauma	0/654(0)	0/181(0)	0/473(0)	
Musculoskeletal/Skin	1/654(0.2)	0/181(0)	0/473(0)	
Transplant	0/654(0)	0/181(0)	0/473(0)	
**APACHE II score**	n=654<br>8 (5-12)	n=181<br>8 (5-11)	n=473<br>8 (4-13)	0.440
**ICU admission source**	n=649	n=181	n=468	0.000*
**Same Hospital**				
ED	256/649 (39.4)	64/181 (35.4)	192/468 (41.0)	
Ward	293/649 (45.1)	89/181 (49.2)	204/468 (43.6)	
Operating theater	10/649 (1.5)	2/181 (1.1)	8/468 (1.7)	
ICU/HDU	46/649 (7.1)	23/181 (12.7)	23/468 (4.9)	
Other Hospital				
ED	10/649 (1.5)	0/181 (0)	10/468 (2.1)	
Ward	25/649 (3.9)	1/181 (0.6)	24/468 (5.1)	
Operating theater	0/649 (0)	0/181 (0)	0 /468(0)	
ICU/ HDU	9/649 (1.4)	2/181 (1.1)	8/468 (1.7)	
**Readmissions, n (%)**	10/654 (1.5)	3/181 (1.7)	7/473 (1.5)	1.000
**Admission type**				0.693

Operative	19/654 (2.9)	4/181 (2.2)	15/473 (3.2)	
Non-operative	635/654 (97.1)	177/181 (97.8)	458/473 (96.8)	
**Emergency surgery**	12/19 (63.2)	3/4 (75.0)	9/15 (60.0)	0.711
**Comorbidities top 15**				
Hypertension	293/654 (44.8)	85/181 (47.0)	208/473 (44.0)	0.461
Diabetes	112/654 (17.1)	38/181 (21.0)	74/473 (15.6)	0.090
Type 2 diabetes	70/654 (10.7)	8/181 (4.4)	62/473 (13,1)	0.001*
Type 1 diabetes	66/654 (10.1)	32/181 (17.7)	34/473 (7.2)	0.000*
Dyslipidemia	39/654 (6.0)	19/181 (10.5)	20/473 (4.2)	0.002*
Hypothyroidism	27/654 (4.1)	12/181 (6.6)	15/473 (3.2)	0.046*
Cardiovascular diseases	20/654 (3.1)	10/181 (5.5)	10/473 (2.1)	0.023*
Renal failure, moderate to severe	15/654 (2.3)	5/181 (3.3)	10/473 (2.1)	0.620
CKD requiring dialysis	13/654 (2.0)	6/181 (3.3)	7/473 (1.5)	0.081
Asthma	12/654 (1.8)	2/181 (1.1)	10/473 (2.1)	0.389
HIV	12/654 (1.8)	4/181 (2.2)	10/473 (2.1)	0.658
Renal failure, Mild	9/654 (1.4)	4/181 (2.2)	5/473 (1.1)	0.654
Rheumatological condition	8/654 (1.2)	4/181 (2.2)	4/473 (0.8)	0.155
Arrhythmia	7/654 (1.1)	3/181 (1.7)	4/473 (0.8)	0.245
Congestive heart failure	6/654 (0.9)	1/181 (0.6)	4/473 (0.8)	0.432

Data is presented as frequency (%) or median (interquartile range)
*classification based on APACHE IV coding.
HDU, high dependency unit; ICU, intensive care unit; ED, emergency department; APACHE, acute physiology and chronic health evaluation; CKD, chronic kidney dysfunction

Supplement Table 7: Clinical condition on admission and main laboratory values for the COVID-19 subgroup

Variables	All patients (n=654)	Patients in HDU (n=181)	Patients in ICU (n=473)	P-value
**Vital signs on admission**	n=652	n=181	n=471	
Systolic blood pressure	125 (114-140)	125 (114-138)	126 (113-140)	0.729
Diastolic blood pressure	74 (65-83)	74 (66-83)	73 (65-84)	0.853
Respiratory rate	22 (19-28)	22 (19-25)	22 (19-28)	0.141
Heart rate	88 (76-101)	85 (75-96)	89 (77-104)	0.008*
Temperature	36.7 (36.5-36.9)	36.6 (36.5-36.8)	36.7 (36.4-37.0)	0.170
**Glasgow coma scale**	n=654<br>15 (14-15)	n=181<br>15 (15-15)	n=473<br>15 (12-15)	0.000*
**AVPU condition**	n=633	n=181	n=452	
Alert	508/633 (80.3)	167/181 (92.3)	341/452 (75.4)	
Sedated	85/633 (13.4)	10/181 (5.5)	75/452 (16.6)	
Unresponsive	10/633 (1.6)	1/181 (0.6)	9/452 (2.0)	
Pain	8/633 (1.3)	0/181 (0)	8/452 (1.8)	
Verbal	22/633 (3.5)	3/181 (1.7)	19/452 (4.2)	
**Blood gas analysis**				
FiO_2,	n=552<br>0.6 (0.4-1.0)	n=159<br>0.6 (0.3-0.9)	n=393<br>0.6 (0.4-1.0)	0.035*
PaO_2	n=443<br>70 (58 - 85)	n=156<br>74 (61.7- 86)	n=287<br>68 (57-85)	0.041*
PaO_2/FiO_2	n=443<br>111 (78-198)	n=156<br>125 (85-236)	n=287<br>105 (74-181)	0.031*
Arterial pH	n=454<br>7.4 (7.4-7.5)	n=156<br>7.4 (7.4-7.5)	n=298<br>7.4 (7.4-7.5)	0.076
**Laboratory**				
Haemoglobin	n=619<br>13.2 (11.7-14.8)	n=180<br>13.1 (11.7-14.6)	n=439<br>13.3 (11.6-14.9)	0.379
Platelet	n=618<br>242 (177-306)	n=180<br>241 (172-307)	n=438<br>243 (180-303)	0.983
Packed cell volume	n=349<br>40.0 (35.8-43.5)	n=116<br>40.1 (36.4-42.6)	n=233<br>39.6 (35-43.6)	0.909
White blood cell count	n=613<br>9.4 (6.7-12.8)	n=180<br>9 (6.4-12.1)	n=433<br>9.5 (6.8-13.2)	0.120
Serum Sodium	n=627<br>137 (133-139)	n=181<br>136 (133-139)	n=446<br>137 (134-140)	0.106
Serum Potassium	n=627<br>4.2 (3.8-4.6)	n=181<br>4.3 (4.0-4.7)	n=446<br>4.2 (3.8-4.6)	0.036*
Serum HCO3	n=417<br>23.9 (21.0-26.8)	n=173<br>23.9 (21.3-26.8)	n=244<br>23.9 (20.7-26.6)	0.662
Serum bilirubin	n=345<br>9 (7-13)	n=111<br>10 (8-14)	n=234<br>9 (7-12)	0.089
Blood urea	n=614<br>5.9 (3.2-9.1)	n=179<br>6.9 (4.3-10)	n=435<br>5.3 (2.6-8.8)	0.000*

Supplement Table 8: Management characteristics for the COVID-19 subgroup

Variables	All patients (n=654)	Patients in HDU (n=181)	Patients in ICU (n=473)	P-value
**Ventilation status on admission**	n=652	n=181	n=471	
Spontaneous	460/652 (70.6)	141/181 (77.9)	319/471 (67.7)	0.000*
HFNT	6/652 (0.9)	2/181 (1.1)	4/471 (0.8)	
NIV	90/652 (13.8)	33/181 (18.2)	57/471 (12.1)	
Invasive ventilation	96/652 (14.7)	5/181 (2.8)	91/471 (19.3)	
**Therapeutics**	n=654	n=181	n=473	
Use of sedatives	75/654 (11.5)	2/181 (1.1)	73/473 (15.4)	0.000*
Use of vasopressors	23/654 (3.5)	3/181 (1.7)	20/473 (4.2)	0.188
RRT	26/654 (4.0)	6/181 (3.3)	20/473 (4.2)	0.587
Antimicrobial use	410/654 (62.7)	66/181 (36.5)	344/473 (72.7)	0.000*

Data is presented as frequency (%) or median (interquartile range)
Abbreviations: ICU, intensive care unit; HDU, high dependency unit; HFNT, high flow nasal cannula; NIV, non invasive ventilation.

Supplement Table 9. Patients outcomes for the COVID-19 subgroup

Variables	All patients (n=654)	Patients in HDU (n=181)	Patients in ICU (n=473)	P-value
**Mortality at ICU discharge**	217/654 (33.2)	38/181 (21.0)	179/473 (37.8)	0.0008*
**Discharge destination for survivors**	n=437	n=143	n=294	0.000*
Ward	328/437 (75.1)	121/143 (84.6)	207/294 (70.4)	
ICU	40/437 (9.2)	11/143 (7.7)	29/294 (9.9)	
Home	21/437 (4.8)	5/143 (3.5)	16/294 (5.4)	
HDU	11/437 (2.5)	0/143 (0)	11/294 (3.7)	
Other hospitals	21/437 (4.8)	6/143 (4.2)	15/294 (5.1)	
Others	12/437 (2.7)	0/143 (0)	12/294 (4.1)	
Transfer for specialist care	4/437 (0.9)	0/143 (0)	4/294 (1.4)	
**Length of stay, days**	n=654<br>6 (3-12)	n=181<br>5 (2-10)	n=473<br>7 (3-13)	0.024*
**Duration of mechanical ventilation, days**	n=102<br>1 (1-12)	NA	n=102<br>4 (1-12)	0.037*
**Left against medical advice***	2/654 (0.3)	1/181 (0.6)	1/473 (0.2)	0.000*
**Discharge upon patient request****	12/654 (1.8)	4/181 (2.2)	8/473 (1.7)	0.000*

*defined as the patient leaving the ICU against the advice of their medical team.
**defined as the decision to discharge from ICU made by a patient and facilitated by the clinical team (e.g. transfer to another hospital at patient's request).
#daily registry data not available in HDU
Abbreviations: ICU, intensive care unit; HDU, high dependency unit
